# Gastric hepatoid adenocarcinoma with multiple liver metastasis: a case report 

**Published:** 2021

**Authors:** Mariano Quino-Florentini, Frank Garcia-Rojas, Pedro Guerra-Canchari, Gustavo Cerrillo-Sanchez

**Affiliations:** 1 *Facultad de Medicina Humana, Universidad Nacional Mayor de San Marcos, Lima, Perú*; 2 *Department of Gastroenterology, * *Hospital Nacional Dos de Mayo, Lima, Perú*; 3 *Sociedad Científica de San Fernando, Lima, Perú*; 4 *Department of Pathology, * *Hospital Nacional Dos de Mayo, Lima, Perú*

**Keywords:** Hepatoid adenocarcinoma, Metastases, Liver

## Abstract

Hepatoid adenocarcinoma is a poorly differentiated alpha-fetoprotein-producing (AFP) tumor frequently located in the stomach, ovary, and pancreas. Presentation in the stomach has a high mortality rate due to late diagnosis, which offers the patient few therapeutic alternatives. On February 22, 2019, a 44-year-old woman from Lima entered the emergency department for pain in the right hypochondrium for 4 months, weight loss, nausea, and asthenia. On physical examination, hepatomegaly presented with a liver spam of 17 cm. Serology showed severe anemia and AFP of 49,800. The tomography showed multiple hypodense lesions in the liver and the presence of nodes. Endoscopy showed Bormann III gastric malignancy. Gastric biopsy determined undifferentiated epithelial malignancy; the immunohistochemical mark (+) for AFP and PAS Diastase confirmed a hepatoid gastric adenocarcinoma. A rare variant of gastric adenocarcinoma was evident, which often mimics an HCC. In this case, multiple liver metastases were observed that differed from the diagnosis of HCC, so this variant must always be taken into account when a primary gastric tumor presents with hepatic metastases.

## Introduction

 Gastric-presenting hepatoid adenocarcinoma is a rare and lethal type of tumor, accounting for less than 1% of all gastric cancers. It belongs to the group of solid tumors of extrahepatic origin that produce alpha-fetoprotein, which can be found in high concentrations in plasma and histologically with immunohistochemical techniques (1). However, its presence in plasma is not decisive, because its serum concentration is related to the presence of metastasis, and therefore, a worse prognosis (2, 3).

AFP-producing gastric cancers are characterized by a high rate of cell proliferation and extensive lymphovascular invasion, due to which approximately 75% metastasize at diagnosis with the liver being the main site (3), and have an estimated overall mean survival of 10 months (4). Among gastric cancers that produce AFP, the hepatoid morphological pattern is the most common (5).

Studies report that AFP-producing gastric adenocarcinoma is histologically classified into three categories: yolk sac, hepatoid, and enteroblastic (5). Among them, the enteroblastic type seems to be associated with the hepatoid type in some tumors. Histologically, these tumors are composed of various proportions of light to slightly eosinophilic tumor cells with tubular, cribriform, papillary, solid, and/or trabecular growth patterns, and immunohistochemical results show positivity for AFP, Hep-Par 1, Glypican-3, and SALL4 (6).

There is insufficient evidence for management, with radical gastrectomy and early-stage adjuvant chemotherapy being the therapy of choice. However, chemo-infusion via the hepatic artery of 5-fluorouracil and cisplatin has successfully increased survival (7). Herein, we report a case of hepatoid gastric adenocarcinoma with a late and confusing diagnosis when presenting multiple liver metastases that simulated hepatocellular carcinoma. 

## Case Report

**Figure 1.**
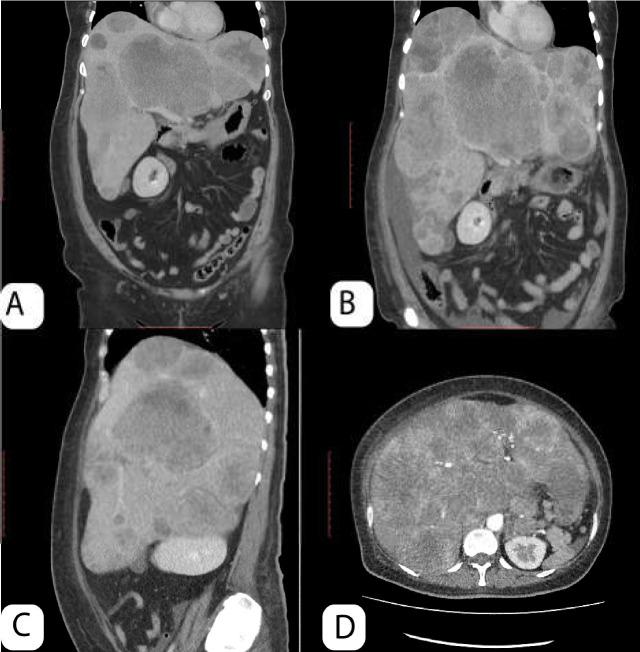
(A) Computed Tomography (CT) at admission shows multiple hypoattenuating liver lesions. (B) CT in coronal plane 2 weeks later with severe hepatomegaly, increasing liver lesions with peripheral rim of enhancement. (C) Sagittal plane demonstrates hepatomegaly fill abdominal cavity and compromises right kidney. (D) Contrast-enhanced computed tomography in arterial phase resembling hepatocellular carcinoma pattern

A 44-year-old female patient with a history of diabetes mellitus under treatment, anemia, and hemorrhoids was admitted to the emergency department for nausea, asthenia, and intense oppressive pain in the right hypochondrium. She reported these symptoms had been worsening for 4 months, and she had experienced a marked weight loss, especially in the last month. On physical examination, hepatomegaly marked with a 17-cm liver spam was found. In addition, an abdominal ultrasound, performed in previous weeks, showed multiple hypodense lesions at the liver level, suspicious for a neoformation. An abdominal tomography was requested to corroborate the ultrasound data. In the emergency room, ranitidine 50 mg, dimenhydrinate 50 mg, and hydration were indicated in addition to a general blood and biochemical examination. The results showed severe anemia (Hb = 7.9 mg/dl), thrombocytosis (platelets = 1,006,000), and an excessive elevation of gamma glutamyl transpeptidase (GGT = 1114 U/L) as well as alkaline phosphatase (FA = 898 U/L). Tomography revealed hepatomegaly with a heterogeneous parenchyma and multiple lobulations (Figure 1). Moreover, stomach wall irregularities were found with abnormal thickening predominantly of the fundus and body and increased mesenteric lymphadenopathy (20 x 19 mm) at the level of the minor curvature as well as in the retroperitoneal paraaortic and iliac nodes. Based on these results, the patient was admitted.

**Figure 2:**
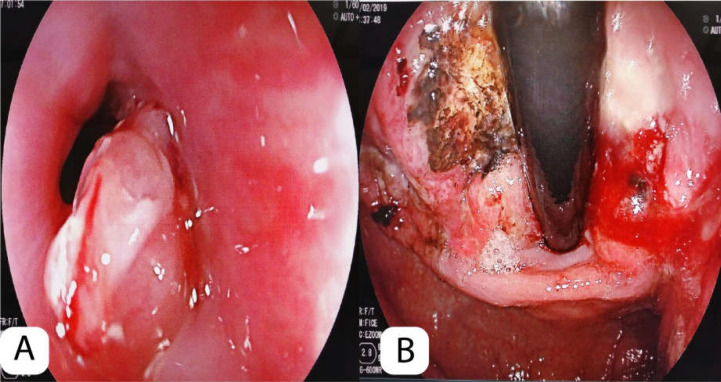
(A) Upper endoscopy found an infiltrative, exofitic, defined borders lesion that narrows and involves the distal portion of esophagus and cardiac. (B) This lesion located in fundus, gastric body and lesser curvature has nodular appearance, 7cm in size with necrotic zones is suggestive of advanced gastric cancer Borrmann III type

**Figure 3:**
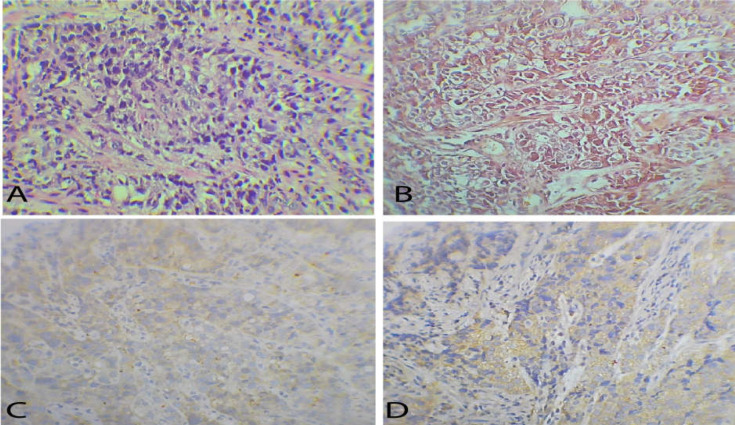
(A): Histopathologic features of gastric biopsy with HE staining shows polygonal hepatoid cells, dismorphic cytoplasm, enlarged nuclei and prominent nucleoli. (B) Trabecular pattern in HE staining and vascular proliferation with poorly differentiation. (C) Hepatoid cells diffusely positive to alpha-fetoprotein. (D) CK7 positive in gastric mucosal

An upper endoscopy was performed to rule out neoplasm. An infiltrative lesion with raised edges was found at the level of the stomach's cardia that extended to the bottom, suggestive of a BORRMANN III-scale malignant neoplasm of the stomach, and a sample was taken for biopsy (Figure 2). Tumor markers showed a slight increase in the CEA and CA 125 markers; however, the Alpha fetus protein (AFP) was at very high levels (AFP = 49800 ng/mL), suggesting the possibility of coexisting gastric neoplasm and primary liver tumor. As a liver biopsy could not be performed, we sought to evaluate the possibility of primary hepatocellular carcinoma by performing triphasic tomography looking for the phenomenon of “wash out”, which is present in most hepatocellular carcinomas. A “wash out” was not evident in this patient, so the possibility of a primary liver neoplasm was ruled out.

One week after admission, another endoscopy was performed with biopsy to confirm the diagnosis of a probable malignant neoplasm of the stomach at the gastric esophageal junction with injury. Infiltrative ulceration with raised edges and a fibrin-covered bed with areas of necrosis that extended through the lesser curvature and posterior wall to the middle 1/3 of the body of the stomach were found. The pathology report mentioned undifferentiated carcinoma of probable gastric origin, undifferentiated carcinoma of probable epithelial lineage. Immunohistochemistry had the following markers: AFP (+ localized), CK7 (+ in gastric-like glands), CK20 (-) synaptophysin, and chromogranin (-) CD45 (+) PAS Diastase (+) (Figure 3). It was concluded to be a hepatoid-type gastric adenocarcinoma. On March 6, the patient presented with a febrile syndrome, so antibiotic therapy was started for probable urinary tract infection (UTI).

Two weeks after admission, the oncology department scheduled four chemotherapy courses with the following scheme: cisplatin, docetaxel, and fluorouracil. The patient completed the first course of chemotherapy; however, after that, she presented with a hydroelectrolytic alteration (hyponatremia) in addition to edema in both legs. The next day the edema became more pronounced, and diuretics were started. One day later, the patient arrived to continue her chemotherapy course, but in the chemotherapy room, she suffered decompensation and died on the way to trauma shock.

## Discussion

Hepatoid adenocarcinoma of the stomach is a rare aggressive tumor, accounting for about 0.8% of all reported gastric cancer cases in China in 2015 (3). It presents pathologically similarly to hepatocellular carcinoma and occasionally produces AFP (2.5). It occurs mostly in the stomach; however, it has also been reported in the lungs (7) and the peritoneal cavity (8). This cancer usually occurs in individuals older than 60 years (about 60%), and has a marked predominance in males (3, 4). The most common gastric clinical presentation is abdominal pain followed by epigastric discomfort with hematemesis and/or melena; the most frequent locations of the tumor in the stomach are the antrum, body, and cardia (9). The present case evidenced a 44-year-old female patient presenting with tumor from the cardia extending to the fundus.

This type of gastric cancer metastasizes through the lymph nodes, and the most common sites of metastasis are the liver followed by the lung (10, 11). Sometimes, however, it cannot metastasize (4). In addition, it has been observed to present with high levels of AFP; about 85% of patients present with levels greater than 40 ng/mL with higher levels seen in males (3). Highly variable levels have been observed in some reports (4). Thus, AFP levels ≥ 500 ng/mL are significantly associated with worse overall survival and tend to be associated with worse disease-free survival (1). When liver metastases exist, they present primarily as liver nodules, and this pathology can be confused with hepatocellular carcinoma in the presence of high levels of AFP (10, 12). In the current case, our patient presented with high levels of AFP and liver metastasis, which is why a probable HCC was suspected; however, the presence of multiple nodules made HCC less likely.

The final diagnosis of hepatoid gastric adenocarcinoma was made through pathology and immunohistochemistry. It has been seen that the vast majority (about 70%) present with AFP (+) (10). Other positive markers found are CEA and certain types of cytokeratins such as CK8, CK18, and CK19. CK7 was found to be positive in about 30%, and CK20 was positive in less than 30% of patients (9, 11). Currently, there are other positive markers such as Glypican 3. In addition, LIN 28 and SALL 4 are highly specific markers to differentiate them from hepatocellular carcinomas, despite the fact that the sensitivity of LIN 28 is lower than that of SALL 4 (13). In the case of our patient, she presented AFP (+), CK7 (+), CK20 (-), CD45 (+) and PAS (+), confirming the diagnosis of hepatoid gastric adenocarcinoma with multiple liver metastases.

There is little evidence of treatment to be offered patients with metastatic hepatoid gastric adenocarcinoma. It was found that chemotherapy of hepatic arterial infusion with 5-fluorouracil (5-FU)/cisplatin (CP) was effective in local cases, and systemic chemotherapy with a regimen including Ramucirumab (RAM) may be effective in cases of metastasis (14). In isolation, the application of cisplatin and etoposide was seen in two metastatic cases; a positive response was seen in one case, further supporting the recommendation of the use of cisplatin (11). The prognosis of these patients is poor, especially in metastatic cases where low survival rates have been observed (3, 9). A 3-year overall median survival of about 7% was seen in patients overall; however, a statistically significantly worse prognosis was seen in patients with metastases and high levels of AFP (3). The present case shows a patient with a disease time of about 6 months and a fatal outcome, probably due to the late diagnosis and late stage of the disease.

In conclusion, we present the case of a patient with gastric cancer and liver metastases that made the diagnosis of hepatoid gastric adenocarcinoma difficult. Thus, it should always be taken into account in the differential diagnosis of gastric tumors with liver involvement.
